# An Optimized Bidirectional Long Short‐Term Memory Model Based on Hyperspectral Analysis of Protein Content in Milk Powder

**DOI:** 10.1002/fsn3.4556

**Published:** 2024-12-10

**Authors:** Wenjing Zhang, Heru Xue, Xinhua Jiang, Jiangping Liu

**Affiliations:** ^1^ College of Computer and Information Engineering Inner Mongolia Agricultural University Saihan District, Hohhot China; ^2^ Key Laboratory of Big Data Research and Application in Agriculture and Animal Husbandry Saihan District, Hohhot China

**Keywords:** BiLSTM‐Attention model, deep learning, non‐destructive testing, protein content, spectral analysis, WOA optimization

## Abstract

Protein content is an important index in the assessment of dairy nutrition. As a crucial source of protein absorption in people's daily life, the quality of milk powder products not only has a deep impact on the development of the dairy industry, but also seriously damages the health of consumers. It is of great significance to find a faster and more accurate method for detecting milk protein content. This paper utilizes the chemical content of milk powder and hyperspectral data as independent variables. By comparing 14 kinds of preprocessing algorithms, the mean‐centered (MC) method is selected to preprocess the data, and then the combined method of competitive adaptive reweighted sampling (CARS) and uninformative variable elimination (UVE) is used to screen the feature wavelength, so as to establish the model and learn the internal dynamic change law of the feature. Furthermore, the Attention mechanism was introduced to assign different weights to bidirectional long short‐term memory (BiLSTM) hidden states through mapping weighting and learning parameter matrix. To reduce the loss of information and strengthen the influence of important information, at the same time, in order to solve the difficult problem of hyperparameter selection of the model, the whale optimization algorithm (WOA) is proposed to optimize the hyperparameter selection of the model. The test results showed that with WOA‐BiLSTM‐Attention model algorithm, the coefficient of determination (*R* ^2^) of 0.9975 and root mean square error (RMSEP) of 0.0337 in comparison with *R* ^2^ and RMSEP values obtained from BiLSTM‐Attention model algorithm, which were higher by 0.799% lower by 56.5%, respectively. This study provides algorithm support and theoretical basis for fast non‐destructive testing based on deep learning algorithm to predict protein content in milk powder.

## Graphical abstract

Whale algorithm to optimize bidirectional long short‐term memory‐Attention prediction model. Wavelength selection using combined method. High precision protein content in milk powder.

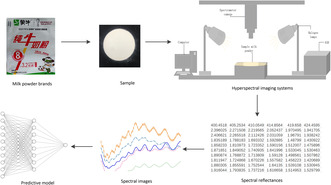

## 1 Introduction

Milk powder is rich source of animal proteins, which can be easily absorbed by the human body and replenish the daily protein requirement of the human body. As an important component of human body, protein is closely related to life (Zou et al. 2016; Guan et al. 2013). Milk powder as a necessary source of protein absorption in people's daily life, its quality problems not only have a deep impact on the development of the dairy industry, so the search for faster and more accurate prediction methods of protein content in milk powder is of great significance to the quality testing of the dairy industry (Feng et al. 2019). The commonly used methods for protein detection are chemical analysis Kjeldahl, ultrasonic analysis, etc. Although the accuracy is high, the process is complex, time‐consuming, and generally requires reagents, which cause damage to the sample and pollute the environment; ultrasonic analysis is sensitive to the ambient temperature. These are no longer able to meet the requirements of rapid, non‐destructive, and accurate food testing.

Hyperspectral imaging (HSI) technology, which refers to the use of high‐resolution spectrometer equipment in many very narrow wavelength bands to receive and record information on electromagnetic radiation reflected or emitted by an object without destroying the object being detected (Li and Zhen 2008). As a new non‐destructive testing technology, the spectral resolution of hyperspectral imaging is in the range of 10^−2^ *λ*. Hyperspectral technology is the result of integrating traditional spectral analysis technology combined with two‐dimensional imaging technology. By capturing the spatial image information and spectral information of the tested sample, the external structure and internal physical and chemical properties can be reflected (Guo and Hui 2015). Hyperspectral imaging technology is characterized by high resolution and a large number of bands (Yu 2020; Huang et al. 2007).

Machine learning models combined with HSI have a wide range of applications in the food field, especially in the prediction of milk composition content. Zhu et al. (2018) constructed a least squares prediction model for the prediction of milk fat content using hyperspectral technique. Mohamed et al. analyzed milk for lactose, protein, and fat content using spectroscopy in the wavelength range of 600–1050 nm. The results demonstrate that spectroscopic techniques can be well applied to the assessment of milk quality (Mohamed et al. 2021). Kasemsumran, Thanapase, and Kiatsoonthon (2007) studied the prediction of water and whey in milk, established soft independent modeling of class analogy (SIMCA) model and discriminant partial least squares (DPLS) model of milk data, and the prediction accuracy reached more than 90%. Carleos et al. demonstrated the potential of the sub‐1000 nm spectral region for milk detection protein and lactose detection of milk spectral data within the range of 400–1000 nm (Carleos et al. 2008; Lu et al. 2009). Qian and Kun (2015) by extracting the hyperspectral image of milk, combining the least square support vector machine (LS‐SVM) and principal component regression (PCR) method, the results show that the prediction accuracy of the model is 0.958. Inácio, Moura, and Lima (2011) proposed a method for classification and determination of total protein in milk powder and used PCR and partial least square regression (PLSR) multivariate calibration to predict protein content.

However, the research methods of milk powder protein detection are mostly similar to traditional machine learning. Considering that deep learning performs well in the field of image processing, some scholars refer to deep learning algorithms in the field of food detection (Medus et al. 2021; Mouazen, Kuang, and Tekin 2015; Cun, Bengio, and Hinton 2015). Ping et al. (2022) used Sparrow algorithm to optimize back propagation neural network (BPNN) algorithm to complete the prediction of milk protein content. Shan et al. (2017) proposed a new bidirectional‐convolutional long‐short term memory (Bi‐CLSTM) network for automatic learning of spectral spatial features of hyperspectral images. The bidirectional recursive connection arithmetic was used to fully mine spectral information. Li et al. (2022) proposed the BiLSTM‐Attention model based on the Attention mechanism to predict atmospheric temperature. By introducing the Attention mechanism, this method solves the limitations in data analysis and improves the accuracy of prediction.

The hyperspectral imaging technology adopted in this paper was used to rapidly detect the protein content of milk powder. The brand milk powder in Inner Mongolia was used as the experimental raw material. These data were collected by hyperspectral imager and the protein content was determined by stoichiometric method as the label. The data of fusion were preprocessed by means of the MC algorithm. A prediction model based on WOA was proposed to optimize BiLSTM‐Attention, and the protein content in milk powder was quantitatively analyzed. The model effectively improves the accuracy of the predicted data, WOA reduces the parameter training time, enhances the ability to jump out of the local optimal, and achieves the global optimal. This method provides theoretical and experimental basis for the application of dairy product quality detection, and has important significance for the protection of consumer rights.

## 2 Materials and Methods

### 2.1 Milk Powder Samples

The experimental samples were obtained from five brands of milk powder in Inner Mongolia. The protein content of each sample is shown in Table 1. These samples used are purchased from local supermarkets. The sample collection container is 90 mm in diameter and 8 mm in height. After opening the can is placed in the container, it is flattened with a flat ruler to make it even. Protein content is shown in Table 1. The content of protein in milk powder was determined by Kjeldahl method, this method is implemented in accordance with the national standard GB5009.5‐2016 of the People's Republic of China. Proteins in milk powder are hydrolyzed under heating conditions with a catalyst, and the resulting ammonia is neutralized with sulfuric acid to produce ammonium sulfate. Alkaline distillation liberates the ammonia, which is titrated with a standard solution of boric acid‐absorbing sulfuric acid, and the nitrogen content is determined based on the consumption of acid, which is then multiplied by a conversion factor to obtain the protein content.

**TABLE 1:** Protein content of milk powder.

Label	Name	Protein content of milk powder (g/100 g)
1	Mengniu High‐calcium	19.0
2	Qishi milk powder	23.8
3	Yanansu goat's milk powder	28.0
4	Mengniu pure milk powder	25.4
5	Shanhe goat's milk powder	20.2

*Note:* The table shows the protein content of five different brands of milk powder brand poles, the protein content in milk powder was determined by Kjeldahl method. The milk powder was purchased from local supermarkets.

In this paper, samples of five different brands of milk powder were collected, 20 samples were taken for each brand, eight areas of interest were selected for each sample through ENVI5.3 software, that is, eight spectral reflectance was obtained. Therefore, the total number of samples was calculated as 5 × 20 × 8 = 800.

### 2.2 Experimental Equipment and Method

**FIGURE 1:**
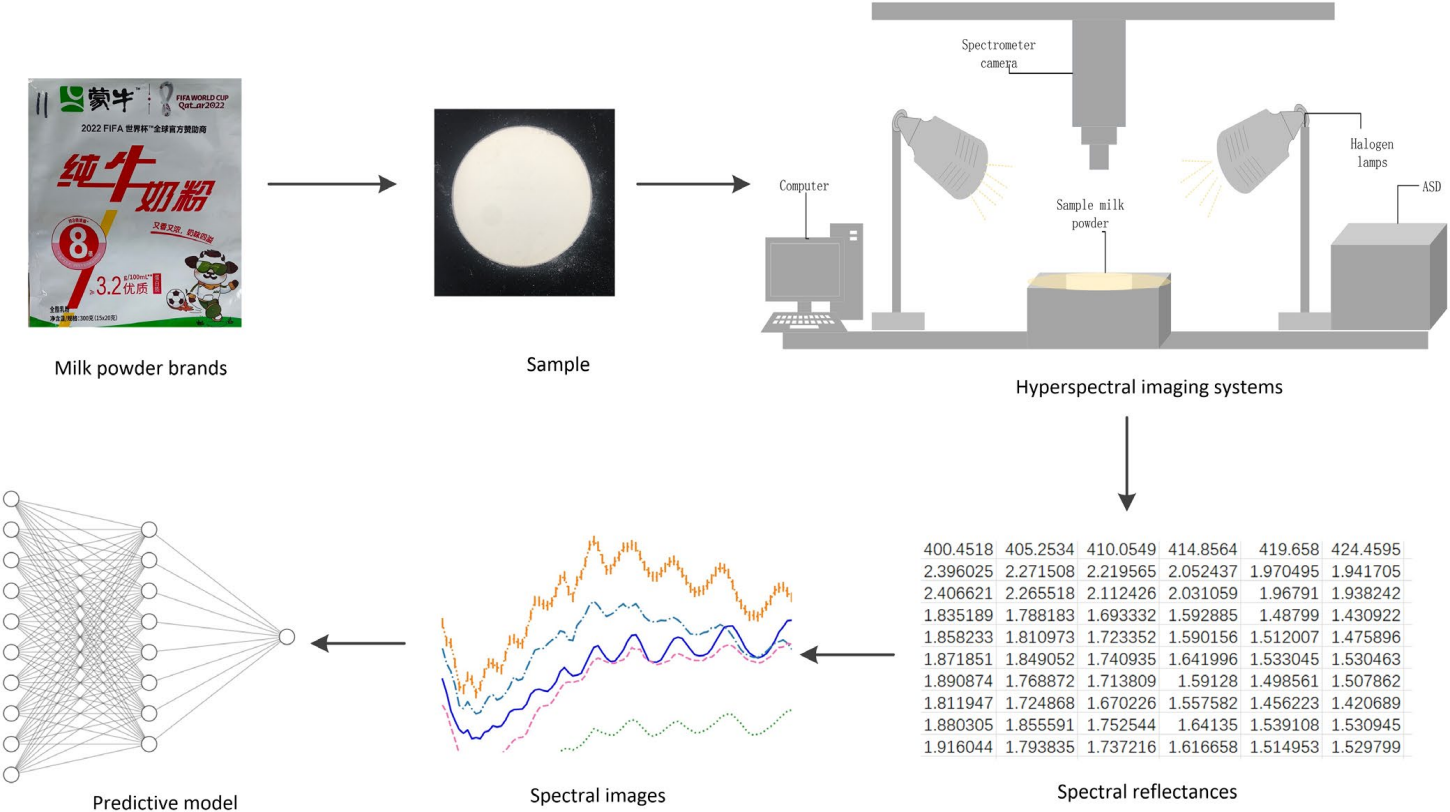
Schematic diagram of a hyperspectral imager. Hyperspectral images of milk samples were acquired using a hyperspectral imaging system. Procurement of milk powder–Production of samples–Acquisition of spectral images by hyperspectral equipment–Spectral reflectance of milk powder in ENVI5.3 software—Spectral reflectance images by python3.6 and Tensorflow2.10.0 framework, and ultimately the prediction model of protein content in milk powder. The hyperspectral imager HyperSpec PTU‐D48E produced by Headwell in 2010 was used as the hyperspectral measurement instrument.

Hyperspectral images were obtained by scanning the surface of milk with hyperspectral imaging system. The hyperspectral acquisition system is composed of a high‐resolution spectral imager, power supply for quartz halogen tungsten lamp lighting, a computer, etc., as shown in Figure 1. The hyperspectral imager HyperSpec PTU‐D48E produced by Headwell in 2010 was used as the hyperspectral measurement instrument. The imaging system has a total of 125 bands, its spectral range is 400–1000 nm, spectral resolution is 4.8 nm, and the resolution of the acquired spectral image is 777 × 1004 pixels.

The flow of the experiment is shown in Figure 1. Hyperspectral images of milk samples were collected by using hyperspectral imaging system. Samples of each type of milk were collected three times, and then a clear image was selected from Environment for Visualizing Images (ENVI5.3) software. ENVI software was used to select areas of interest from hyperspectral images and derive the spectral reflectance of milk powder (Munir et al. 2018). The spectral reflectance images were derived by python3.6 and Tensorflow2.10.0 frames, and the prediction model of protein content in milk powder was finally established. All experiments were performed at a suitable humidity of 35% ± 2% and temperature of 24°C ± 1°C.

In the process of spectral acquisition, the sensor in the spectral camera will produce dark current, and the spectral data collected each time will be accompanied by certain noise information, which will affect the quality of the hyperspectral image. Therefore, in order to reduce the influence of objective factors, black and white correction of the system is required before image acquisition (Zhou et al. 2014). Black and white correction is performed according to Formula (1). Among them is the corrected spectral image of milk powder, the spectral image of milk powder collected during the experiment, the all‐black corrected spectral image after the camera cover is covered, and the all‐white corrected spectral image after the camera cover is removed.

(1) $$I_{c}=\frac{I_{r}-I_{b}}{I_{w}-I_{b}}$$

### 2.3 Experimental Analysis

#### 2.3.1 Data Preprocessing

Milk powder is a kind of mixed solid powder; the main components are protein, fat, calcium, carbohydrates, etc. In the spectral information collected, in addition to the required basic sample characteristics, often also doped with unnecessary irrelevant information and noise, such as stray light, strong electrical noise, artificial noise in the transmission process, etc. Spectral pretreatment methods mainly include the following:

Data enhancement transform: Enhance the difference between the spectra of the samples to improve the stability and prediction ability of the model. Such as MC, StandardScaler (SS), and MinMaxScaler (MMS). Smoothing method: A method that reduces the random error carried by itself and improves the signal‐to‐noise ratio by averaging spectral information data, such as convolution smoothing method—Savitzky–Golay (S–G) (Savitzky and Golay 1964). Derivative method: The derivative algorithm can eliminate the interference caused by baseline drift or flat background, distinguish overlapping peaks, and improve resolution and sensitivity, such as first‐order derivatives (1stDer) and second‐order derivatives (2ndDer) (Min et al. 2016).

**FIGURE 2:**
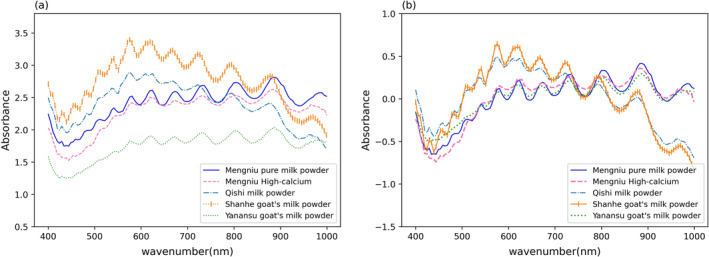
(a) Spectral curves of original milk powder sample; (b) spectral profile of milk samples after MC pretreatment. The spectral reflectance image of milk powder is shown in (a), which shows the spectral data of five brands of milk powder. (b) shows the spectral image after MC preprocessing. MC, mean centering method.

In this paper, different pretreatment methods are compared, and different pretreatment methods are combined to match the auxiliary pretreatment. Through experimental comparison, the pretreatment method suitable for the spectral data is selected. The spectral reflectance image of milk powder is shown in Figure 2a, which shows the spectral data of five brands of milk powder. Figure 2b shows the spectral image after MC preprocessing.

#### 2.3.2 Characteristic Wavelength Selection Based on CARS Combined With UVE

As the hyperspectral data have the characteristics of multiple wavelengths and high dimensionality, the characteristic wavelength selection method can eliminate the noise band, reduce the number of cycles, simplify the model structure, and improve the model performance.

Researchers have compared several commonly used feature wavelength selection methods, such as CARS, continuous projection algorithm (SPA), UVE, and genetic algorithm (GA), and found that CARS has the best effect (Gen et al. 2018). CARS is a feature variable selection method that combines Monte Carlo sampling with regression coefficient of PLS model (Shuang et al. 2014). CARS algorithm can not only preferentially select characteristic wavelength, but also eliminate redundant wavelength, which to a large extent overcomes the problem that too many combinations must be considered when selecting variables. Therefore, selecting an optimized subset of variables can also improve the predictive of the model.

Sun Tong et al. used UVE preferential selection method to detect chromium content in soybean oil (Tong et al. 2016). The core of the UVE algorithm is to select the characteristic wavelength of the spectrum itself by using statistics of variable information unrelated to noise. The most significant point is to use or add noise. The UVE model employs cross‐validation to evaluate the significance of each variable by introducing an equal number of white noise variables as the original variables in the PLS model. For each variable, the stable values of the regression coefficients, that is, the mean of the regression coefficients divided by their standard deviation, were computed and compared with the stable values of the random noise variables, a criterion used to identify and exclude variables that contribute little or nothing to the model predictions, thus filtering out useful characteristic variables (Guo, Gu, and Liu 2016).

In order to select a stable feature wavelength, the number of iterations is increased, and the feature wavelength generated by each iteration is selected based on its significant contribution. Out of 125 characteristic wavelengths, 65 wavelengths with the highest contribution rate were selected by CARS algorithm, and then 53 characteristic wavelengths of milk powder spectrum were selected by UVE algorithm. By employing feature wavelength screening, the time complexity of prediction model can be reduced while enhancing its predictive capability.

### 2.4 Recurrent Neural Network Prediction Model

#### 2.4.1 BiLSTM Predictive Model

LSTM neural networks are suitable for processing and predicting important events with very long intervals and delays in time series. LSTM is a special type of recurrent neural network (RNN), which overcomes the disadvantages of RNN gradient disappearance and difficulty in maintaining long‐term memory (Sherstinsky 2020; Christian, Patrick, and Kai 2021). The principle adopted by LSTM in information prediction involves inferring information from both preceding and subsequent data, only considering the feature relationship of the current moment, without capturing the relationship between the front and back information. Therefore, based on one‐way LSTM, BiLSTM is proposed, which considers both the earlier and later information.

BiLSTM is a combination of forward LSTM and backward LSTM. It was first proposed by Jurgen Schmidhuber and Sepp Hochreiter in 1997. It combines forward LSTM and reverse LSTM to obtain more complete context information. Therefore, BiLSTM can better capture bidirectional semantic dependencies (Yu et al. 2019). BiLSTM is computed as forward and backward computation, where the horizontal axis represents the bidirectional flow of time and the vertical axis represents the unidirectional flow of information from the input layer to the hidden layer and the hidden layer to the output layer.

**FIGURE 3:**
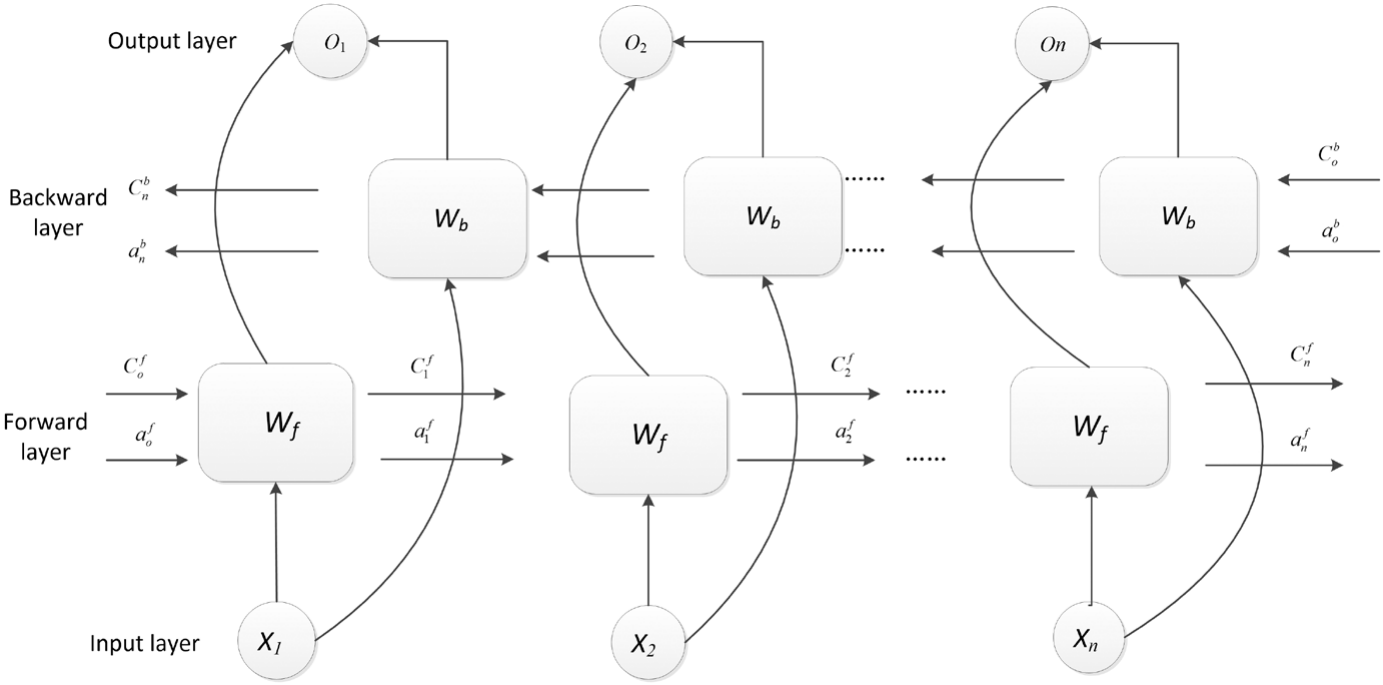
BiLSTM network structure diagram. The BiLSTM model is shown in this figure: at each moment, the output results of the forward layer and the reverse layer are combined at the corresponding time to obtain the final output, which combines the information of the bidirectional input sequence. The forward hidden vector is computed separately ${\vec{h}}_{t-1}$ generates a new implicit vector ${\vec{h}}_{t}$ and backward implicit vectors ${\overset{\leftarrow }{h}}_{t+1}$ generates a new implicit vector ${\overset{\leftarrow }{h}}_{t}$. The results are obtained by combining the output results of the positive and negative input sequences *O*_*t*_. *W*_f_ represents the forward LSTM layer. *W*_b_ represented as a backward LSTM layer. *b*_*y*_ represents an offset item. BiLSTM, bidirectional long short‐term memory neural networks; LSTM, long short‐term memory neural networks.

The BiLSTM model is shown in Figure 3: the forward hidden vector is computed separately ${\vec{h}}_{t-1}$ generates a new implicit vector ${\vec{h}}_{t}$ and backward implicit vectors ${\overset{\leftarrow }{h}}_{t+1}$ generates a new implicit vector ${\overset{\leftarrow }{h}}_{t}$. The results are obtained by combining the output results of the positive and negative input sequences $O_{t}$.

In the Forward layer of the previous layer, the forward calculation is performed from time 1 to time *t*, and the output of the forward hidden layer at each time is obtained and saved. *W*_f_ represents the forward LSTM layer.

(2) $${\vec{h}}_{t}=\text{LSTM}\left(x_{t},{\vec{h}}_{t-1}\right)$$

Compute backward from time *t* to time 1 in backward layer to obtain and save the output of the backward hidden layer at each time, *W*_b_ represented as a backward LSTM layer.

(3) $${\overset{\leftarrow }{h}}_{t}=\text{LSTM}\left(x_{t},{\overset{\leftarrow }{h}}_{t+1}\right)$$

At each moment, the output results of the forward layer and the reverse layer are combined at the corresponding time to obtain the final output, which combines the information of the bidirectional input sequence. $W_{\vec{h}y}$, $W_{\overset{\leftarrow }{h}y}$: a weight matrix representing each connection to the previous hidden state, *b*_*y*_ represents an offset item.

(4) $$O_{t}=\tanh\left(W_{\vec{h}y}{\vec{h}}_{t}+W_{\overset{\leftarrow }{h}y}{\overset{\leftarrow }{h}}_{t}+b_{y}\right)$$

The forward and reverse networks in BiLSTM are independent, and their outputs are spliced together as inputs for the next layer. In the training process, the parameters of the forward and reverse network are shared parameters. The stacking of two layers of LSTM enables the model to better combine the output of the information before and after, and the model is robust without too much dependence on the features. However, the disadvantages of this model are: BiLSTM is not strong enough in feature extraction, and the output splicing method of forward and backward fusion features is not good enough, thus necessitating the incorporation of an Attention mechanism.

#### 2.4.2 Attention Mechanism Assignment of Weights

The Attention mechanism is a resource allocation mechanism that simulates the Attention of human brain. At a certain moment, the human brain will focus its Attention on the areas that need to be focused, reduce or even ignore the Attention to other areas, so as to obtain more details that need Attention and suppress other useless information. The core idea is to change the Attention to information skillfully and reasonably. Ignore irrelevant information and amplify useful information (Lei et al. 2023). Through probability allocation, sufficient Attention is allocated to key information to emphasize the impact of important information, thereby enhancing the accuracy of the model.

#### 2.4.3 WOA Optimization—BiLSTM‐Attention Prediction Model

Whale Optimization Algorithm (WOA) is a new swarm intelligence optimization algorithm proposed by Mirjalili and Lewis from Griffith University in Australia in 2016. WOA is a meta‐heuristic optimization algorithm to simulate humpback whale hunting behavior. Compared with other swarm optimization algorithms, WOA is a meta‐heuristic optimization algorithm. WOA employs random or optimal search agents to simulate hunting behavior, and spirals to simulate humpback whales' bubble‐net attack mechanism (Mirjalili and Lewis 2016). WOA algorithm characterized by its simple mechanism, minimal parameters, strong optimization ability, etc., and has been widely used in economic scheduling, optimal control, image segmentation and other aspects (Gang et al. 2023).

WOA optimized BiLSTM prediction model based on Attention mechanism. The model utilized hyperspectral data and protein content in milk powder as input data, captured the internal dynamic change rule of learning features, and incorporated Attention mechanism to give different weights to BiLSTM hidden states through mapping weighting and learning parameter matrix. Reducing the loss of historical information and enhancing the influence of important information is referred to as BiLSTM‐Attention prediction model. Additionally, due to the challenges in selecting hyperparameters for the model, the WOA algorithm has been proposed to optimize this process.

**FIGURE 4:**
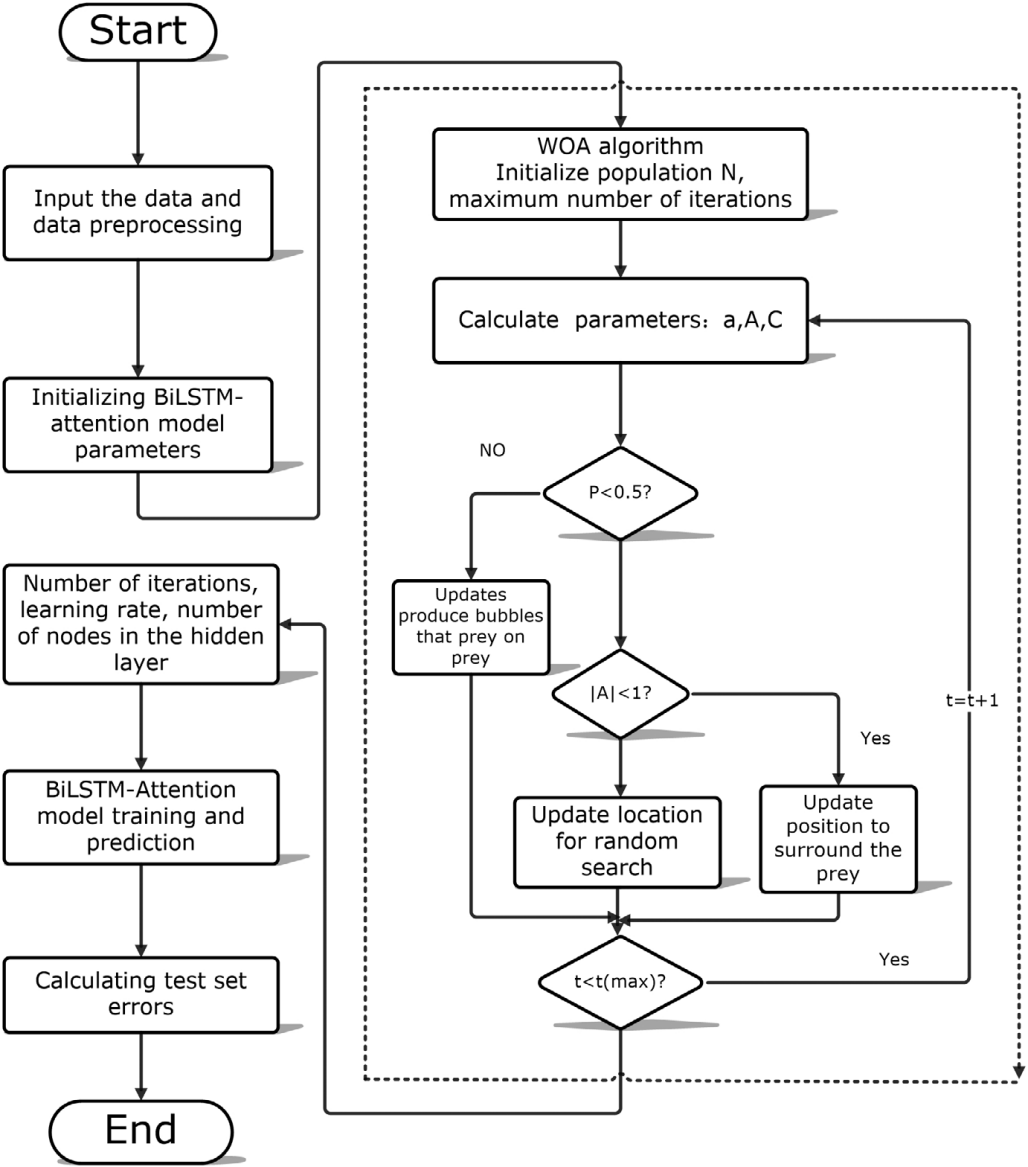
Predictive model flow chart, where the dashed box shows iterative flowchart of whale optimization algorithm. WOA optimization is the problem of finding parameter optimization. Minimizing the mean square error between the expected and actual outputs of the BiLSTM‐Attention network is taken as the fitting function, which is, finding a set of network hyperparameters that minimize the error. The whole optimization process is shown in this figure, where the dotted line is the WOA algorithm process.

The WOA optimization problem involves finding the maximum or minimum value of the fitness function. In this paper, the mean square error between the expected output and the actual output of the BiLSTM‐Attention network is minimized as the fitness function, that is, a set of network hyperparameters is found to minimize the error. The entire optimization process is illustrated in Figure 4, where the dashed line is the WOA algorithm process:

1. Input the spectral data and preprocessing from start.
2. The BiLSTM‐Attention component first decodes the parameters provided by WOA to determine the number of iterations, learning rate, and the number of nodes of each hidden layer, then uses the training set passed in by the data part to carry out network training, and finally predicts the test set to obtain the mean square error of the actual output value and the expected output value. The mean square error is returned to the WOA algorithm as a fitness value.
3. In the WOA algorithm part, the algorithm moves the finder, follower and alert according to the fitness value, so as to update the population and the global optimal solution. The WOA algorithm process includes three important stages: round up prey, bubble net prey, and search prey:
  - Round up the prey: It is assumed that the current best candidate solution is the location of the target prey or close to the target prey.
(5) $$D=\left|C•X^{*}\left(t\right)-X\left(t\right)\right|$$
(6) $$\;X\left(t+1\right)=X^{*}\left(t\right)-A•D$$
t indicates the number of current iterations, *A* and *C* are coefficient vectors, X*(*t*) is the location of the best solution. *X*(*t*) is the position. Throughout the entire iteration, the value of decreases linearly from 2 to 0; *r*1 and *r*2 are random vectors in [0, 1].

(7) $$A=2a\times r_{i}-a$$

(8) $$C=2\times r_{2}$$

b Bubble net predation: Update relative position with prey by logarithmic spiral equation. This equation is expressed by the Formula (9):
(9) $$X\left(t+1\right)=D^{\prime }\times e^{\mathrm{bl}}\times \cos\left(2\mathrm{\pi l}\right)+X^{*}\left(t\right)\;D^{\prime }=\left|X^{*}\left(t\right)-X\left(t\right)\right|$$
$D^{\prime }$ is the distance between the current search individual and the current optimal solution, *b* is the spiral shape parameter, *l* is the range of [−1, 1]. As there are two kinds of predation behavior in the process of getting close to the prey, the position update Formula (10). In Formula (10), p is a random number with the value range [0, 1]. As the number of iterations *t* increases, the parameter *A* and the convergence factor *a* gradually decrease. If $\left|A\right|<1$, then each whale gradually surrounds the current optimal solution, that is, the local optimization stage.

(10) $$X\left(t+1\right)=\left\{\begin{array}{c} X^{*}\left(t\right)-A\cdot D;p\leq 0.5\\ D\times e^{\mathrm{bl}}\times \cos\left(2\mathrm{\pi l}\right)+X^{*}\left(t\right)p\leq 0.5 \end{array}\right.$$

c Search for prey: The WOA updates the location of the whales based on their distance from each other for the purpose of random search. When |*A*| ≥ 1, the searchers swam to random whales, *D*″ represent the distance between the currently searched individual and the random individual, $X_{\mathrm{rand}}\left(t\right)$ represent the selected location of the current population.

(11) $$D^{\prime \prime }=\left|C•X_{\mathrm{rand}}\left(t\right)-X\left(t\right)\right|$$

(12) $$X\left(t+1\right)=X_{\mathrm{rand}}\left(t\right)-A•D$$

### 2.5 Evaluation Metrics for Predictive Models

In this paper, root mean square error (RMSE), determination coefficient (*R* ^2^), and relative percent deviation (RPD) are used to evaluate the stability and accuracy of the model.

1. Root mean squared error (Chai and Draxler 2014)

(13) $$\;\text{RMSE}=\sqrt{\frac{\sum_{i=1}^{n}{\left(y_{i}-{\hat{y}}_{i}\right)}^{2}}{n}}$$

2 Coefficient of determination (R‐Squared)

(14) $$\begin{array}{c} \;R^{2}=1-\frac{\sum {\left(y_{i}-{\hat{y}}_{i}\right)}^{2}}{\sum {\left(y_{i}-{\bar{y}}_{i}\right)}^{2}}\; \end{array}$$

3 Relative percent deviation

(15) $$\mathrm{RPD}=\frac{1}{\sqrt{1-R_{P}^{2}}}$$

n represents the number of predicted samples, y represents the true value of protein content in milk powder. $\hat{y}$ is the predicted value of protein content of milk powder, and $\bar{y}$ is the average of the true value of protein content of milk powder. The $R^{2}$ range [0, 1] represents the degree of fitting between the predicted value and the true value, taking into account the difference between the predicted value and the true value as well as the degree of dispersion of the true value. The larger the $R^{2}$, the better the model fits. The smaller the RMSE value, the more accurate the model prediction (Chen et al. 2022). When RPD is greater than 2.0, it indicates that the model has good prediction ability (Lan et al. 2022).

## 3 Results and Discussion

### 3.1 Hyperspectral Data Analysis and Data Preprocessing

There are 800 samples in this paper, which are divided into training set and test set according to 7:3 ratio. These spectral data are preprocessed and combined with LSTM model for regression prediction. The LSTM model is used here, which is more suitable for deep learning model than PLSR and nonlinear support vector machine regression (SVR) model.

The results are shown in Table 2. Compared with single preprocessing methods, the method with MC preprocessing (MC‐LSTM model), although $R_{C}^{2}$ = 0.9599 and RMSEC = 0.1349 in the training set, compared with SS‐LSTM model, $R_{C}^{2}$ = 0.9652 and RMSEC = 0.1335 in the training set are 0.549% lower and 1.05% higher, respectively. However, on the test set MC‐LSTM model improves $R_{P}^{2}$ by 0.641% and reduces RMSEP by 12.78% than SS‐LSTM model, and overall MC‐LSTM is better than SS‐LSTM model. MC‐LSTM model $R_{P}^{2}$ = 0.9421 and RMSEP = 0.1617 in the test set are the best among all the prediction models. It shows that the fitting ability of data through MC‐LSTM model is stronger and more stable. In the combined preprocessing model, the SS‐S‐G‐LSTM model performs better. The SS method controls the data dimension within a certain range, and S–G fits the data trend. The two methods play a complementary role, but compared with MC‐LSTM, $R_{C}^{2}$ and $R_{P}^{2}$ decrease by 0.71% and 0.14%, and the training and fitting ability are slightly lower. Therefore, MC is chosen as the preprocessing method.

**TABLE 2:** Results of LSTM prediction by different preprocessing methods.

Predictive models	Training set		Test set	
	$R_{C}^{2}$	RMSEC	$R_{P}^{2}$	RMSEP
MC‐LSTM	0.9599	0.1349	0.9421	0.1617
SS‐LSTM	0.9652	0.1335	0.9361	0.1854
1stDer‐LSTM	0.8919	0.2233	0.8664	0.224
2ndDer‐LSTM	0.838	0.2682	0.8028	0.2945
MMS‐LSTM	0.8615	0.2743	0.8589	0.2754
MC‐S‐G‐LSTM	0.9416	0.1644	0.9318	0.1728
MC‐1stDer‐LSTM	0.8965	0.2185	0.8725	0.2385
MC‐2ndDer‐LSTM	0.8265	0.279	0.7692	0.3193
SS‐S‐G‐LSTM	0.9531	0.1006	0.9407	0.1106
SS‐1stDer‐LSTM	0.8828	0.2325	0.8557	0.2536
SS‐2ndDer‐LSTM	0.8305	0.2758	0.7757	0.3148
MMS‐S‐G‐LSTM	0.9202	0.1477	0.9135	0.1501
MMS‐1stDer‐LSTM	0.7237	0.357	0.6822	0.3764
MMS‐2ndDer‐LSTM	0.9687	0.0977	0.9325	0.1435

*Note:* Comparing 14 preprocessing methods, preprocessing methods with long short‐term memory (LSTM) neural networks to build predictive models. The MC‐LSTM model was shown to have the best prediction through this table, so MC was chosen as the preprocessing method.

Abbreviations: 1stDer, first‐order derivatives method; 2ndDer, second‐order derivatives method; MC, mean centering method; MMS, MinMaxScaler method; S–G, Savitzky–Golay smoothing; SS, StandardScaler method.

### 3.2 Characteristic Band Selection Results and Analysis

**FIGURE 5:**
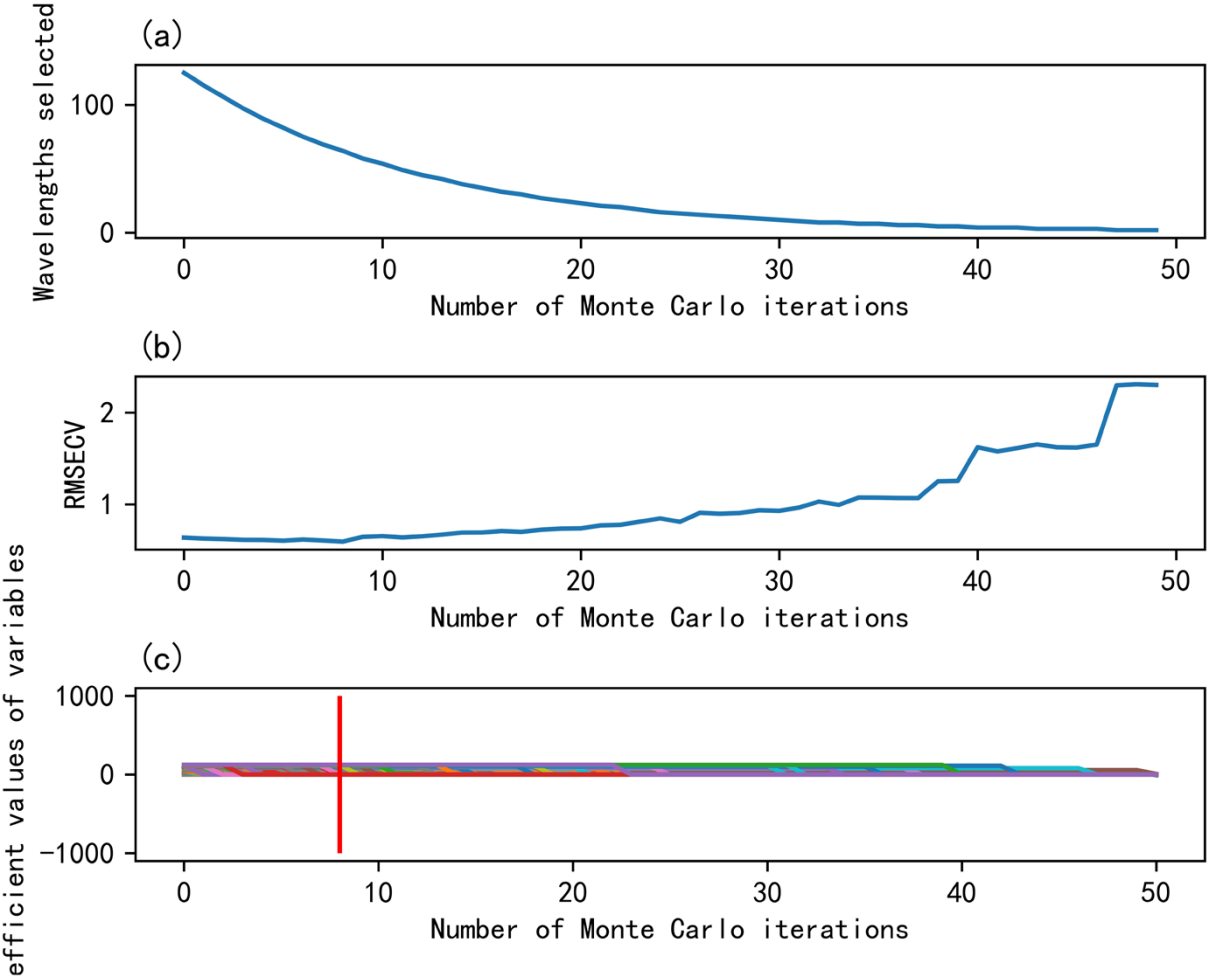
Feature selection process of CARS. Characteristic wavelength of milk powder sampling. (a) Number of sampling variables, which gradually decreases with the increase of the number of runs. (b) Root‐mean‐square error for cross‐validation. With the increase of the number of samples, the RMSECV value of the cross‐validated single PLS model first decreases and then increases, and reaches the minimum value when the number of iterations is 48. (c) Regression coefficient. The red line indicates the lowest RMSECV value. CARS, competitive adaptive reweighted sampling method.

There are more characteristic wavelengths than spectral data, which increases the complexity of predicting space and time for quantitative models. Therefore, the selection of characteristic wavelengths can reduce the number of characteristic wavelengths and improve the efficiency of the model. The wavelength selection of spectral data in data set was carried out by combining CARS and UVE algorithm. The original 125 wavelengths of hyperspectral data were selected by CARS algorithm (in which Monte Carlo sampling times were set to 50, optimal iteration times were 48, and cross‐validation times were 10), and finally 64 wavelengths were selected. The experimental results are shown in Figure 5. Characteristic wavelength of milk powder sampling, Figure 5a number of sampling variables, which gradually decreases with the increase of the number of runs. Figure 5b root mean square error for cross‐validation. With the increase of the number of samples, the RMSECV value of the cross‐validated single PLS model first decreases and then increases, and reaches the minimum value when the number of iterations is 48. Figure 5c regression coefficient. The red—line indicates the lowest RMSECV value.

CARS method screened out half of the original wavelength of the feature wavelength, and then UVE method again screened out 53 feature wavelengths to further reduce the number of feature wavelengths, and combined with LSTM method to predict the model, experimental comparison results are shown in Table 3: CARS‐UVE denoted as CU methodology, MC‐CU‐LSTM model outperforms MC‐CARS‐LSTM in both training and prediction sets. The MC‐CU‐LSTM prediction model compares with the MC‐LSTM model in Table 2, which improves $R_{P}^{2}$ by 2.84% and reduces RMSEP by 28.5% on the prediction set. Therefore, choosing the CU methodology as the feature screening method can effectively improve the model prediction ability.

**TABLE 3:** Comparison of prediction model results was established by combining CARS with UVE(CU) and LSTM.

Predictive models	Training set		Test set		RPD
	$R_{C}^{2}$	RMSEC	$R_{P}^{2}$	RMSEP	
MC‐CU‐LSTM	0.9745	0.1084	0.9682	0.1156	3.9974
MC‐CARS‐LSTM	0.9687	0.1204	0.9552	0.1399	3.3795

*Note:* The table shows the comparison of different feature wavelength selection methods with long short‐term memory (LSTM) neural networks after MC preprocessing. By comparing the data, the CU characterization method can improve the fitting ability and stability of the prediction model.

Abbreviations: CARS, competitive adaptive reweighted sampling method; CU, combining CARS with UVE method; MC, mean ceterning method; UVE, uninformative variable elimination method.

### 3.3 Predicted Results and Analysis of Protein Content of Milk Powder

After MC preprocessing and CU characteristic wavelength selection, the hyperspectral data samples were respectively compared with classical prediction models such as principal component analysis (PCA), PLSR, SVR (Yu et al. 2019; Lei et al. 2023), and compared with BiLSTM, BilSTM‐Attention and WOA‐BiLSTM‐Attention models. After comparison of six prediction models, the experimental results are shown in Table 4. The following conclusions can be drawn from the experiment:

1. PCA‐PLSR, PLSR, and SVR are the traditional spectral data prediction models. PCA proposes two main features from the original 125 characteristic wavelengths and combines them with PLSR to form a PCA‐PLSR model. Compared with the MC‐CU‐PLSR and MC‐CU‐SVR models, the $R_{P}^{2}$ = 0.9584 and RMSEP = 0.6727 of the MC‐CU‐PLSR model are superior to the other two models on the prediction set, but the $R_{P}^{2}$ of the MC‐CU‐LSTM model is lower by 1.02% and RMSEP is 82.8% higher.
2. Comparing the MC‐CU‐BiLSTM model with the MC‐CU‐LSTM model, the BiLSTM algorithm has better fitting effect on the test set than the LSTM algorithm. In addition, in the BiLSTM‐Attention model, with the addition of Attention mechanism and the redistribution of weights, the model is better than BiLSTM in the prediction effect, with an increase of 1.19% in $R_{P}^{2}$ and a decrease of 27.6% in RMSEP in the prediction set.
3. WOA was used to optimize the parameters of BiLSTM‐Attention model: learning rate(lr), max_iterations, batchsize, num_epochs, the number of nodes implied by two‐layer BiLSTM, and dense. The upper and lower bounds on the search optimization for the initial parameter settings of WOA are as follows: max_iterations = 10, lr = [0.01, 0.1], num_epochs = [10, 100], batchsize = [16, 128], hidden1 = [1, 20], hidden2 = [1, 20], Dense = [1, 100]. Parameter after WOA optimization: lr = 0.00829, num_epochs = 83, batchsize = 60, hidden1 = 4, hidden1 = 12, Dense = 60, Attention Layer = 24.

**TABLE 4:** Comparison of effects of predictive models.

Predictive models	Parameters	Training set		Test set		RPD
		$R_{C}^{2}$	RMSEC	$R_{P}^{2}$	RMSEP	
PCA‐PLSR	n_components = 10, max_iter = 10	0.967	0.6024	0.9558	0.6935	3.4013
MC‐CU‐PLSR	n_components = 3	0.9679	0.5943	0.9584	0.6727	3.5026
MC‐CU‐SVR	kernel = ‘rbf’	0.9223	0.9321	0.9146	0.9411	2.4728
MC‐CU‐BiLSTM	num_epochs = 20, batch_size = 16, lr = 0.01, hidden1 = 10, hidden2 = 10, Dense = 12	0.98	0.0952	0.9755	0.105	4.5461
MC‐CU‐BiLSTM‐Attention	num_epochs = 30, batch_size =16, lr = 0.01, hidden1 = 10, hidden2 = 10, Attention Layer = 20, Dense = 20	0.9897	0.0687	0.9872	0.076	6.2696
MC‐CU‐WOA‐BiLSTM‐Attention	lr = 0.00829, num_epochs = 83, batchsize = 60, hidden1 = 4, hidden2 = 12, Dense = 60, Attention Layer = 24	0.9976	0.0327	0.9975	0.0337	14.1565

*Note:* The comparison of the six predicted models is shown in this table. After MC preprocessing and CU characteristic wavelength selection, the hyperspectral data samples were respectively compared with classical prediction models such as PCA‐PLSR, PLSR, and SVR and compared with BiLSTM, BilSTM‐Attention, and WOA‐BiLSTM‐Attention models. The results show that the MC‐CU‐WOA‐BiLSTM‐Attention model is the best trained and fitted, and the optimized parameters avoid the effect of local optimization.

Abbreviations: BiLSTM, bidirectional long short‐term memory; PCA, principal component analysis; PLSR, partial least square regression; SVR, support vector machine regression; WOA, whale optimization algorithm.

**FIGURE 6:**
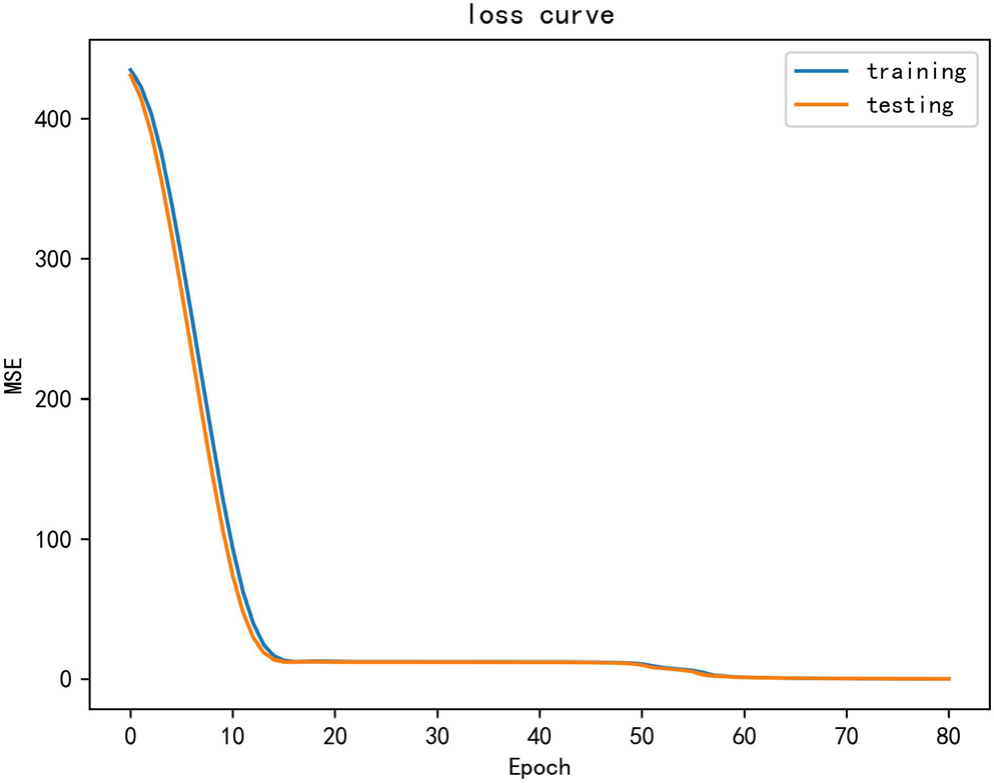
WOA‐BiLSTM‐Attention loss_curve. This figure shows the loss variation curves for the training and prediction sets. With the gradual increase in the number of iterations, the MSE gradually decreases. When num_epochs = 83, the real and predicted values tend to stabilize, and the real and predicted values are basically corresponding, indicating that the model has strong stability. Note: The blue line represents the loss curve for the training set and the yellow line represents the loss curve for the test set.

After WOA optimization of BiLSTM‐Attention model, compared with BiLSTM‐Attention algorithm, $R_{C}^{2}$ increased by 0.799%, RMSEC decreased by 56.5%, $R_{P}^{2}$ increased by 1.04%, and RMSEP decreased by 61.4% in training set. The results show that the model is the best in training and fitting, and the optimized parameters avoid the effect of local optimization. Figure 6 shows the loss evolution curve on the training set and the prediction set. With the gradual increase in the number of iterations, MSE gradually decreases. When num_epochs = 83, the true value and predicted value tend to be stable, and the true value and predicted value are basically corresponding, indicating that the model has strong stability.

## 4 Conclusion

In this study, hyperspectral imaging technology was used to detect protein content in milk powder. The non‐destructive detection of protein content in milk powder was conducted using spectral reflection data. After comparing 14 pretreatment methods, including single and combination pretreatment method, the MC method was selected to preprocess the original data based on its effectiveness in improving the accuracy of model prediction. The combined method of CARS and UVE was used to select the reflectance corresponding to the characteristic wavelength as the independent variable in the model for predicting the protein content of milk powder. The experiment proved that the characteristic wavelength selected by this method could improve the prediction effect of the model. The experiment compared six prediction models: PCA‐PLSR, PLSR, SVR, BiLSTM, BiLSTM‐Attention, WOA‐BiLSTM–Attention, and other prediction models, on the basis of BiLSTM‐Attention model, the WOA algorithm was introduced to determine hyperparameters, in order to avoid local optimization. Moreover, the model demonstrates a stronger fitting ability and the highest prediction accuracy, while also exhibiting a certain level of stability. The WOA‐BiLSTM‐Attention model combined with hyperspectral technology provides a new solution for non‐destructive testing of protein content in milk powder. This prediction model can be utilized for regression analysis in various spectrums, and has great application prospects for the subsequent component identification.

## Author Contributions

**Wenjing Zhang:** conceptualization (lead), methodology (lead), writing – original draft (lead). **Heru Xue:** formal analysis (equal), funding acquisition (supporting), resources (lead). **Xinhua Jiang:** funding acquisition (equal), project administration (lead). **Jiangping Liu:** data curation (equal), funding acquisition (supporting), resources (equal).

## Conflicts of Interest

The authors declare no conflicts of interest.

## Data Availability

The data that support the findings of this study are available on request from the corresponding author. These data are not publicly available due to privacy or ethical restrictions.
